# Supplementary figures and images for: Tuning social interactions’ strength drives collective response to light intensity in schooling fish
Source: PLoS Comput Biol. 2023 Nov 17;19(11):e1011636. doi: 10.1371/journal.pcbi.1011636 (PMC10691717; doi:10.1371/journal.pcbi.1011636)

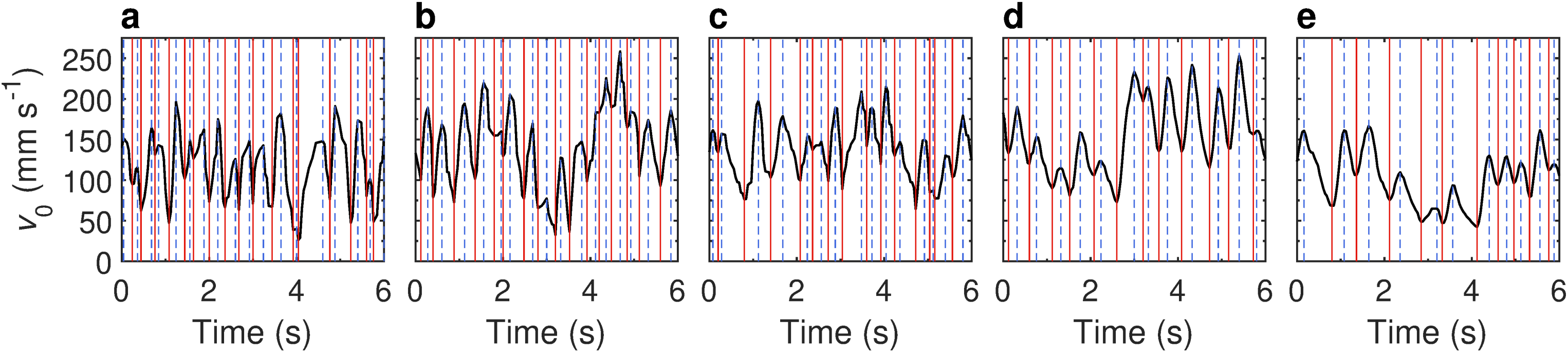

Supplement: S1 Fig — Time series of the instantaneous speed of one fish under different light intensities: a 0.5, b 1, c 1.5, d 5 and e 50 lx. Colored vertical lines represent local minima (red) and maxima (blue) of the speed. Time intervals going from a red line to the next blue line correspond to the bursting acceleration phase, intervals going from a blue line to the next red line correspond to the decelerating gliding phase. (PDF) [file pcbi.1011636.s021.pdf]

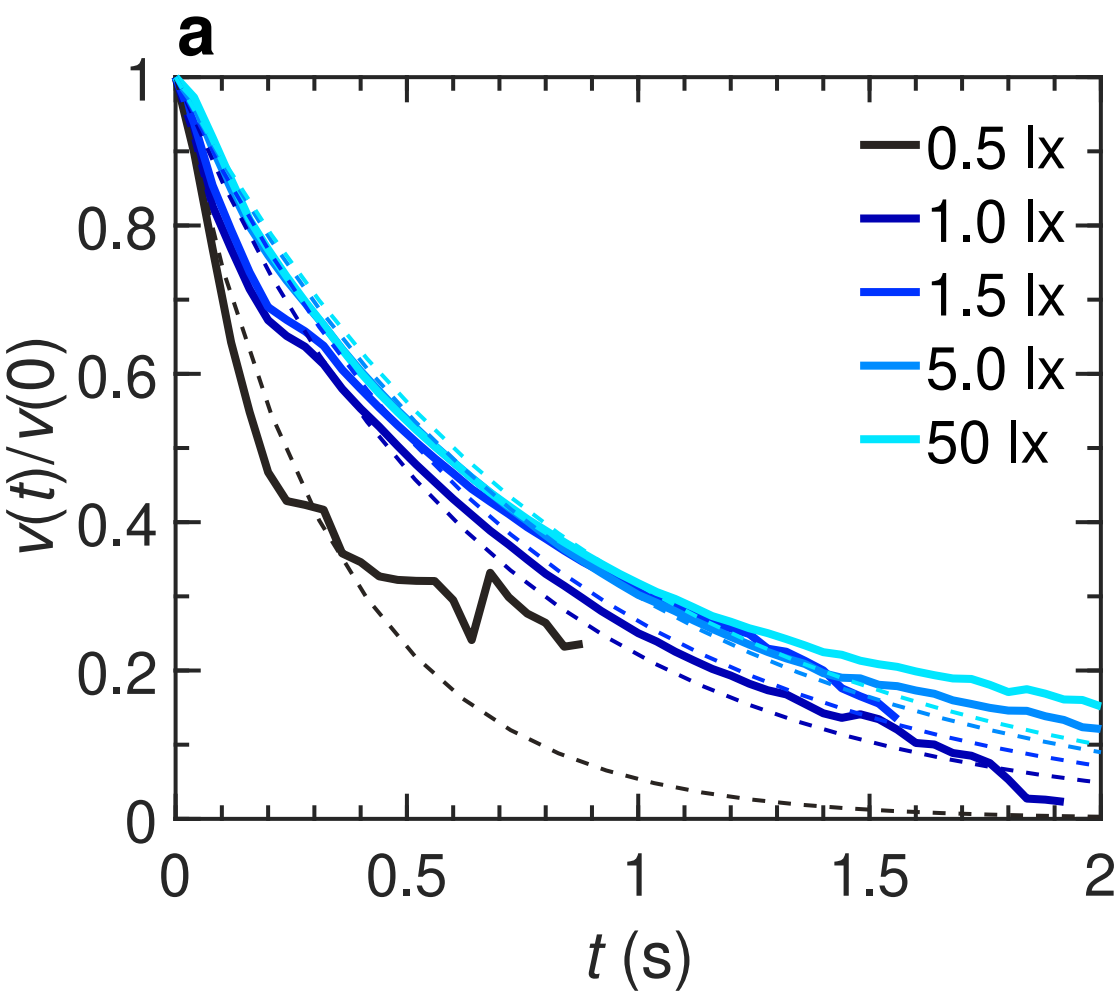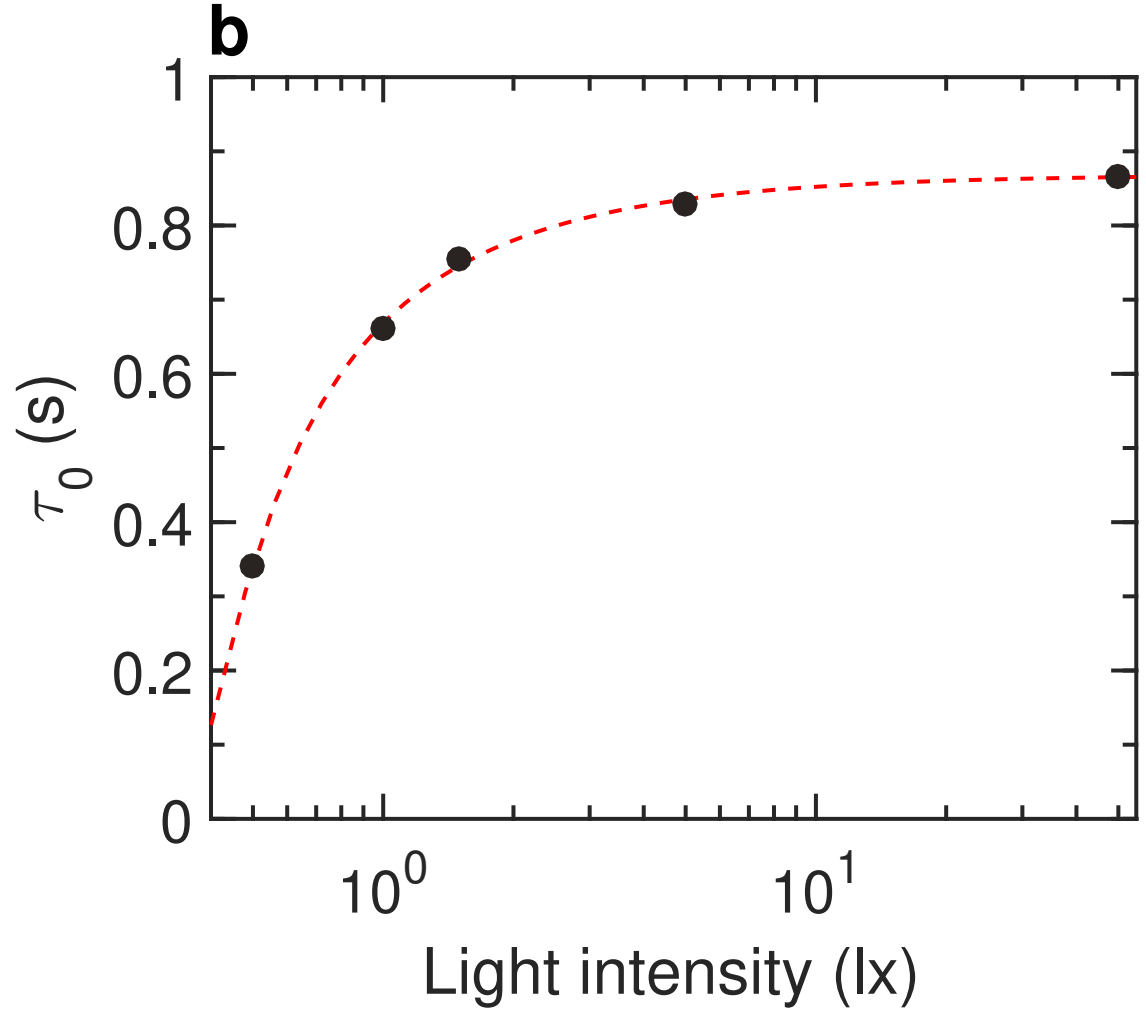

Supplement: S2 Fig — a Exponential deceleration during the gliding phase averaged along all kicks and normalized with the value of the speed at the kicking instant, for different light intensities 0, 0.5, 1, 5, and 50 lx (from dark to light blue). Wide solid lines are experimental measures, dashed lines are exponential approximations of the form exp(−t/τ0), where τ0 is the relaxation time: τ0 ≈ 0.34 (0.5 lx), 0.66 (1 lx), 0.76 (1.5 lx), 0.83 (5 lx), 0.87 (50 lx). b Mean relaxation time τ0 as a function of the light intensity (black circles). The red dashed line shows the trend of the average value with the light intensity. (PDF) [file pcbi.1011636.s022.pdf]

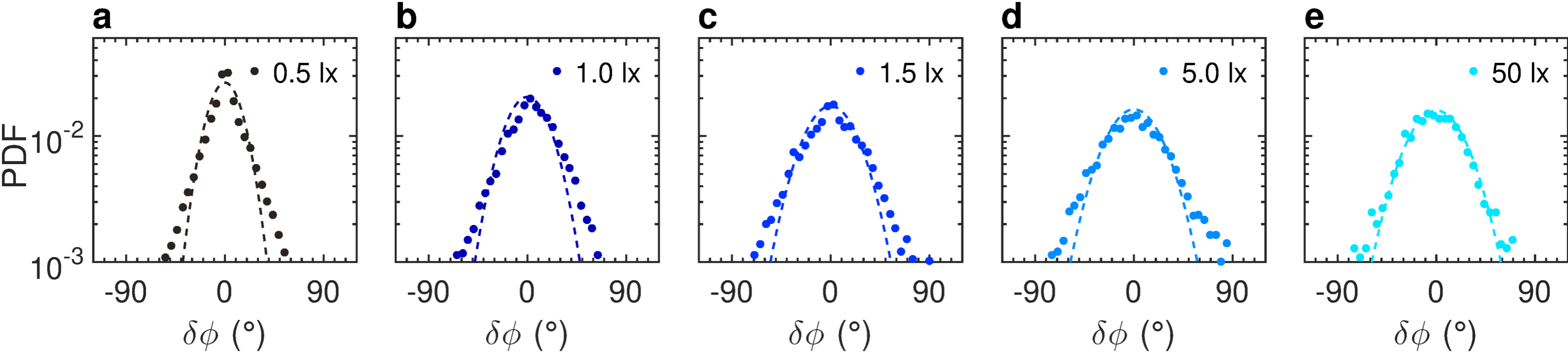

Supplement: S3 Fig — Probability density function (PDF) of the angle variation δϕ when the fish is far from the wall (rw > 60 mm) in five different light intensities: 0.5, 1, 1.5, 5 and 50 lx (from dark to light blue). Colored dots: measures from the experiments. Dashed lines: approximation with Gaussian distributions, with γR=0.26, 0.34, 0.40, 0.42, and 0.43 respectively. (PDF) [file pcbi.1011636.s023.pdf]

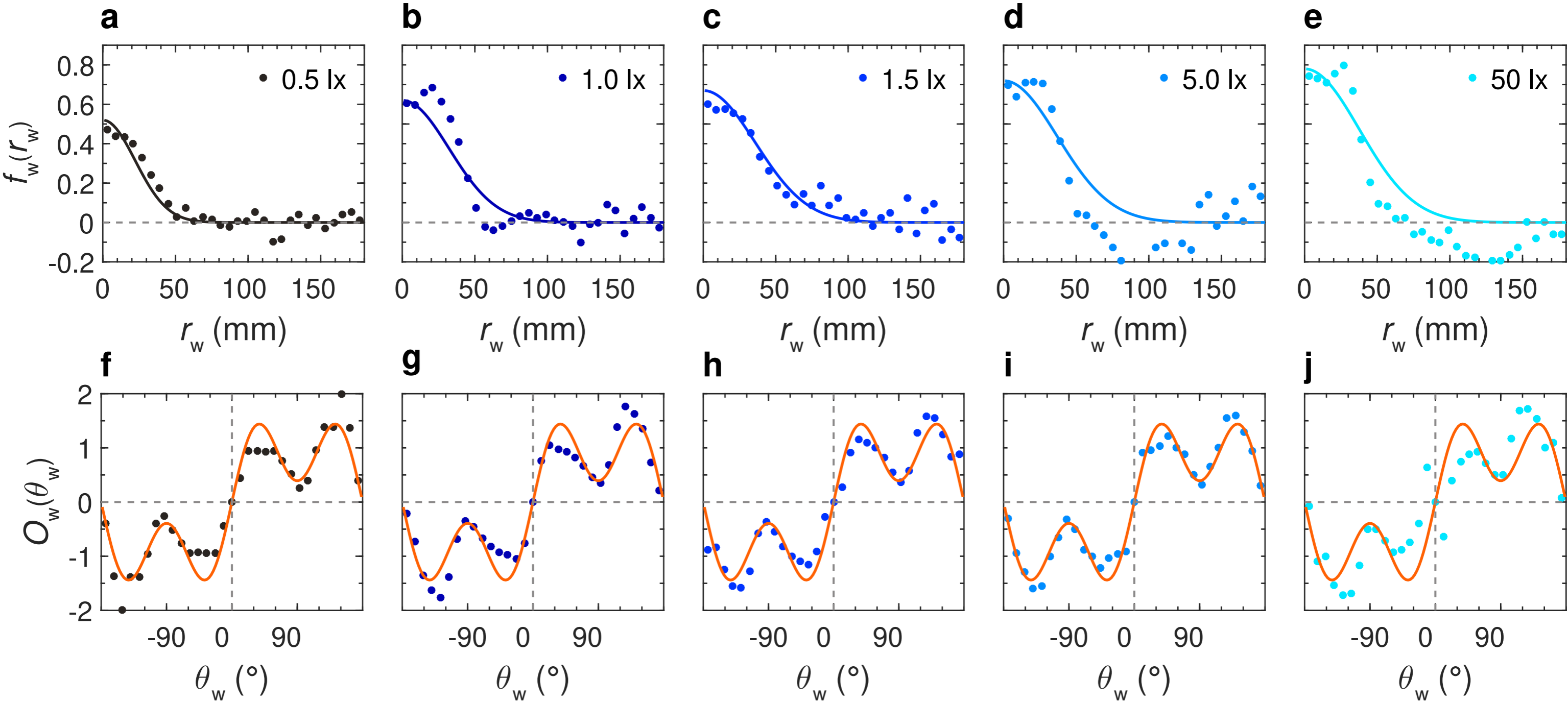

Supplement: S4 Fig — Function of repulsion fw(rw)Ow(θw) as extracted from the experiments by means of the reconstruction procedure (dots), and analytical approximations used in the numerical simulations (solid lines), for different light intensities: 0.5, 1, 1.5, 5, and 50 lx (from dark to light blue). a-e Intensity of the interaction fw(rw) as a function of the fish distance to the wall rw. f-j Intensity of the interaction Ow(θw) as a function of the relative orientation of the fish to the wall θw. Orange lines correspond to the analytical approximation of a single discrete function combining all light conditions: Ow(θw) = 1.9612 sin(θw)[1 + 0.8 cos(2θw)]. (PDF) [file pcbi.1011636.s024.pdf]

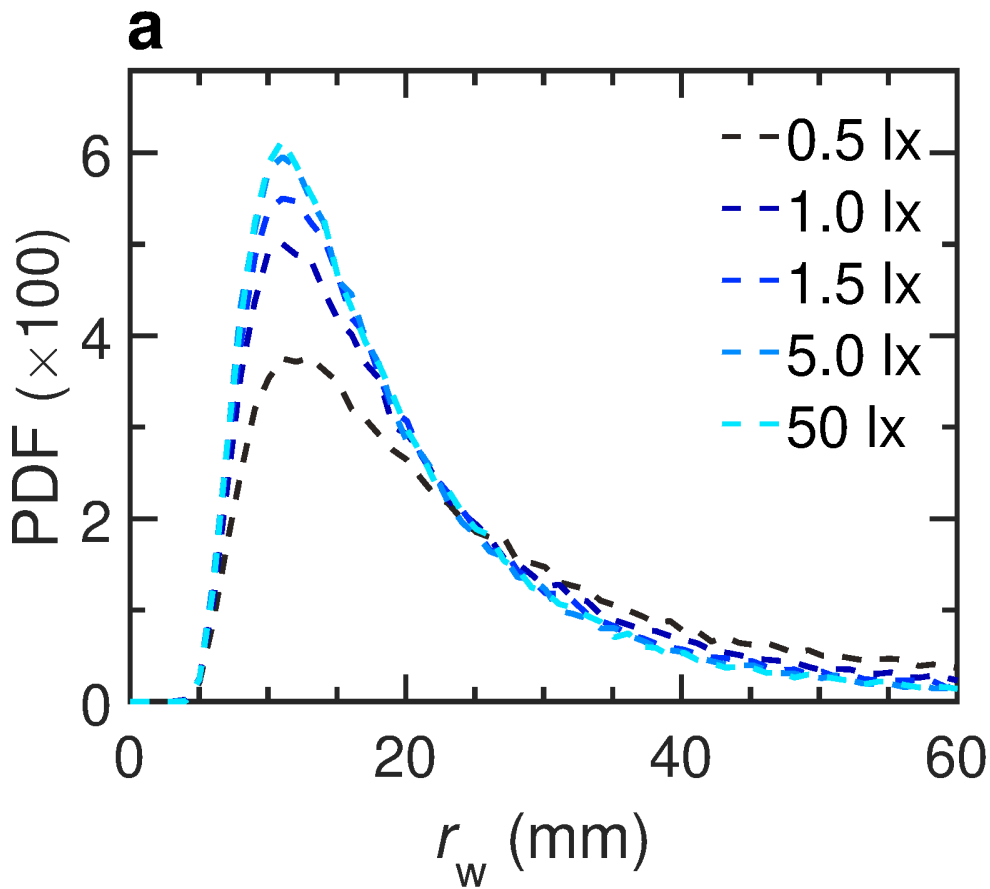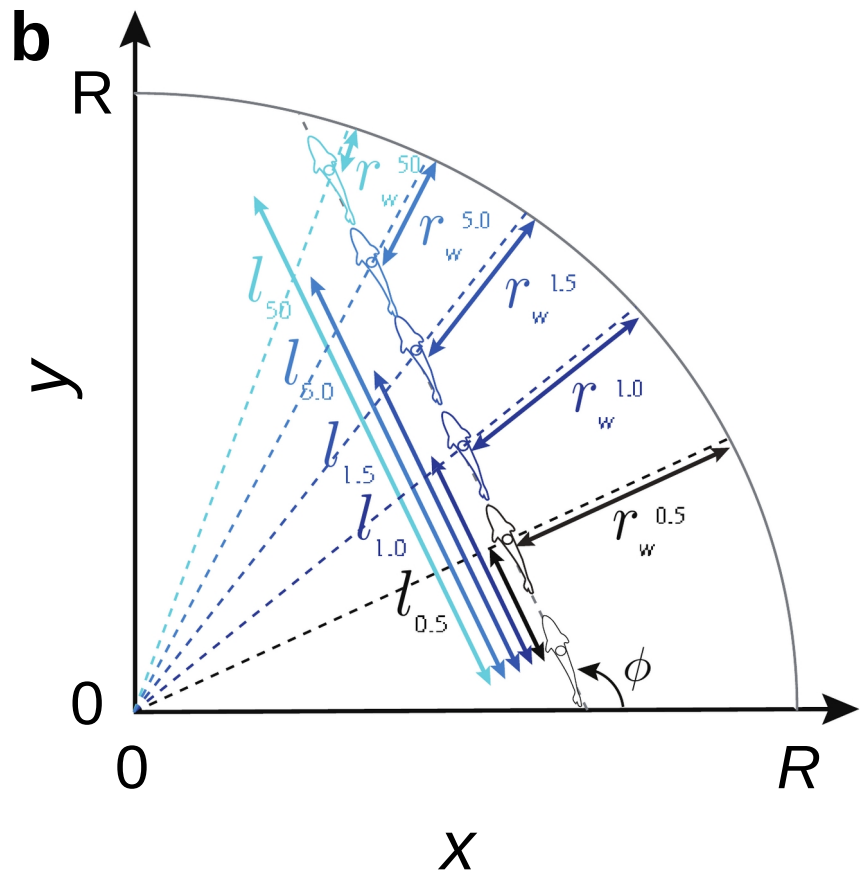

Supplement: S5 Fig — a Probability density function (PDF) of the distance of the fish to the wall rw as a function of light intensity when only the kick length l is changed in the model. b Schematic diagram of the motion of a single fish between two kicks. The distance travelled by the fish between two kicks is greater at 50 lx than at 0.5 lx; as a consequence, the fish moves closer to the wall when the light intensity is higher. (PDF) [file pcbi.1011636.s025.pdf]

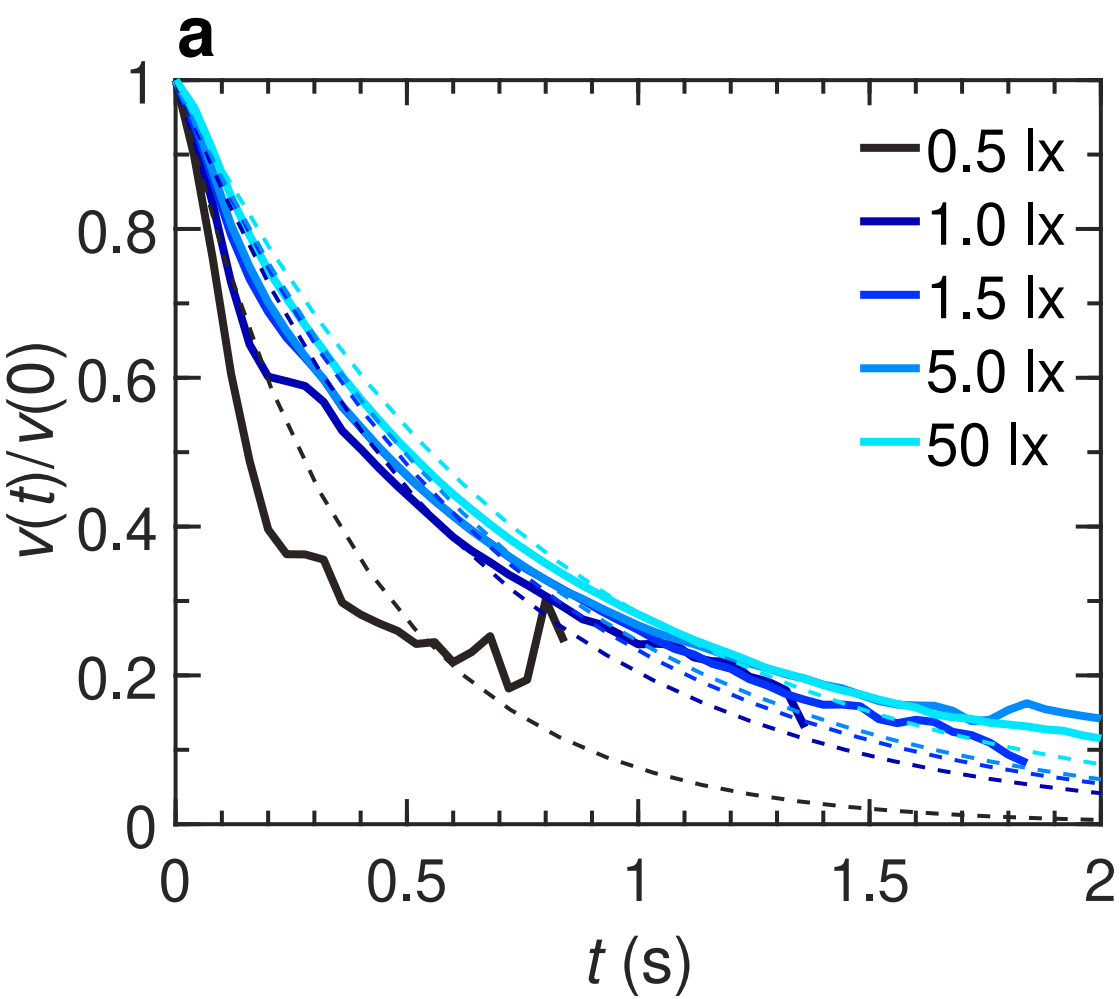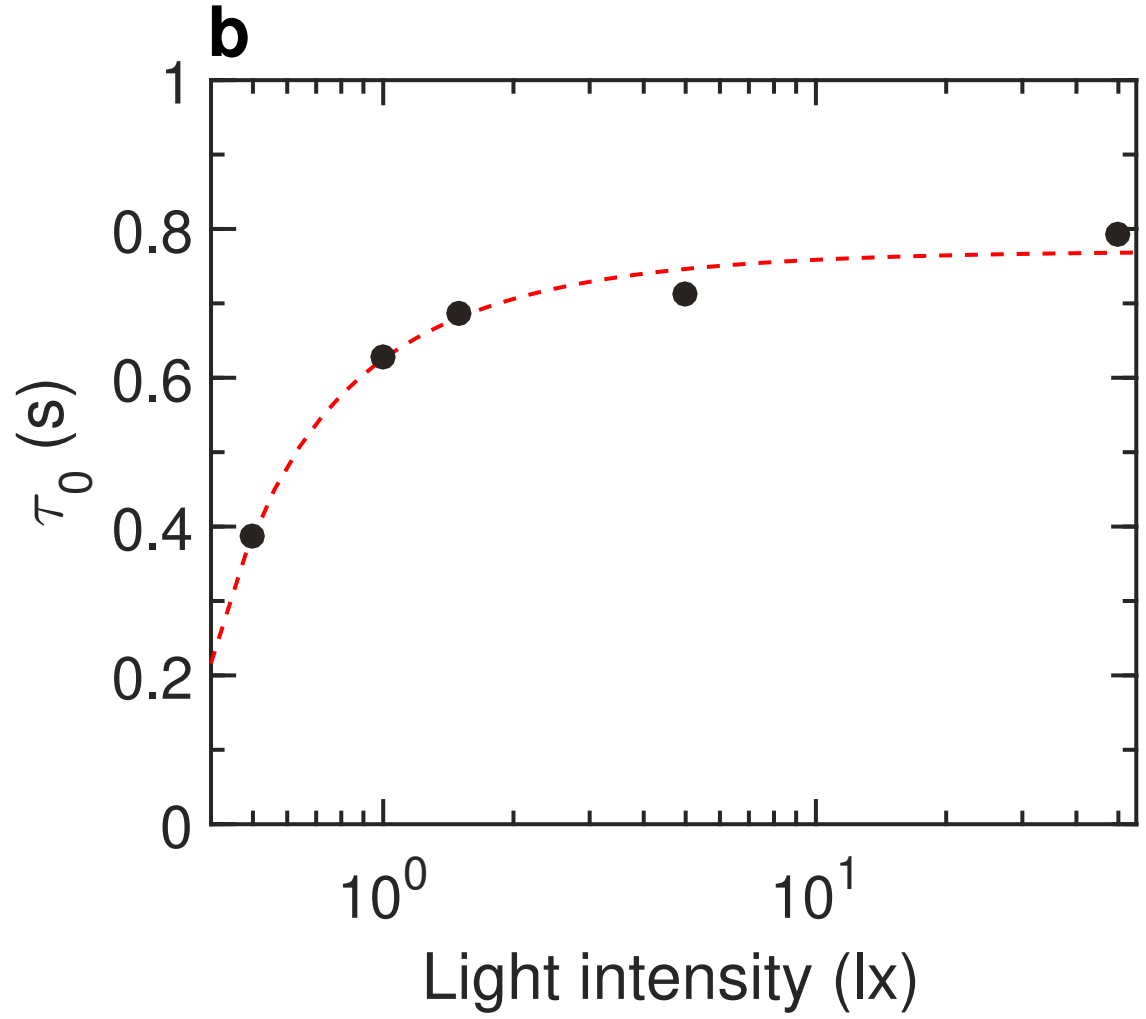

Supplement: S6 Fig — a Exponential deceleration during the gliding phase averaged along all kicks and normalized with the value of the speed at the kicking instant, for different light intensities 0, 0.5, 1, 5, and 50 lx (from dark to light blue). Wide solid lines are experimental measures, dashed lines are exponential approximations of the form exp(−t/τ0), where τ0 is the relaxation time: τ0 ≈ 0.39 (0.5 lx), 0.63 (1 lx), 0.69 (1.5 lx), 0.71 (5 lx), 0.79 (50 lx). b Mean relaxation time τ0 as a function of the light intensity (black circles). The red dashed line shows the trend of the average value with the light intensity. (PDF) [file pcbi.1011636.s026.pdf]

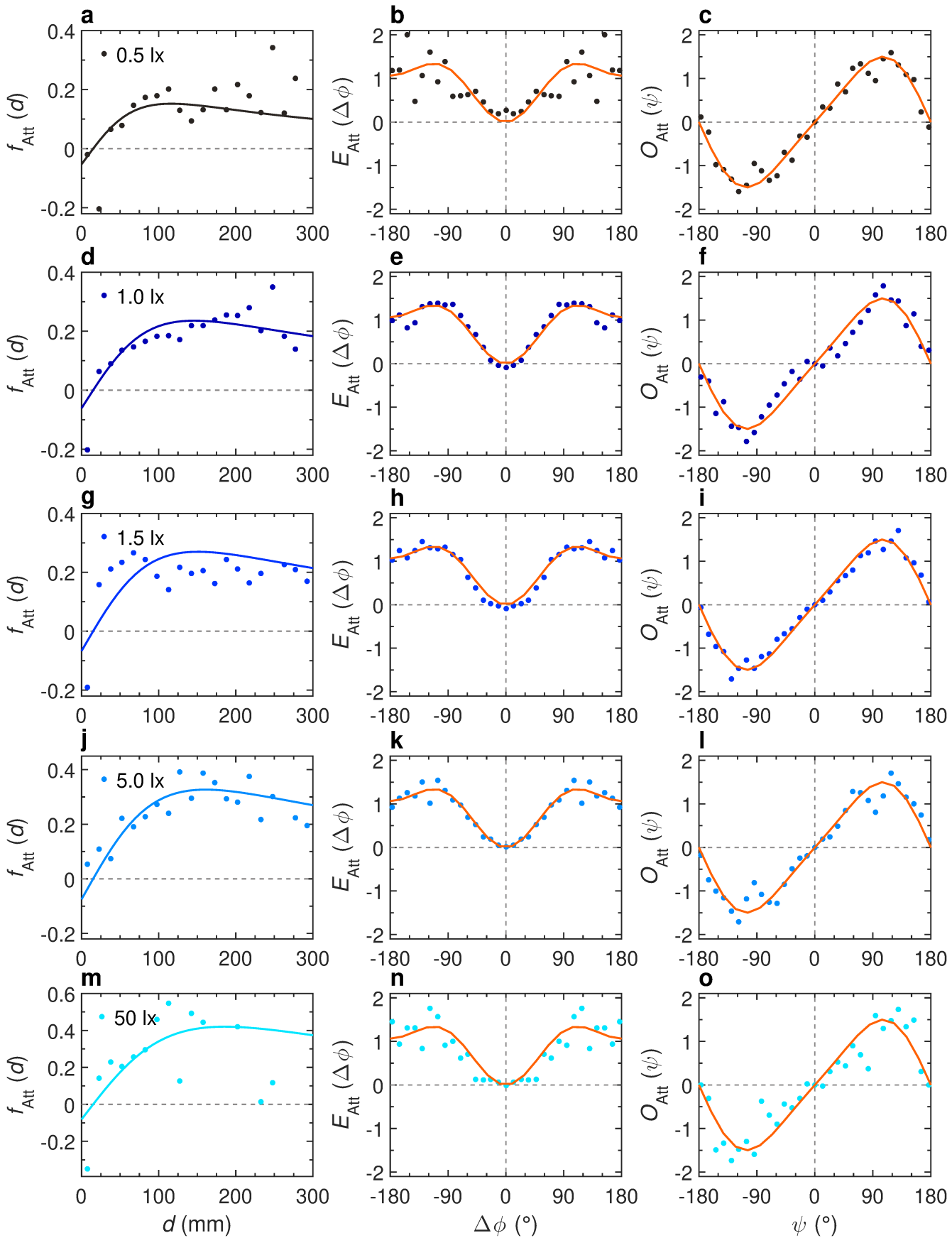

Supplement: S7 Fig — Components of the attraction interaction function fAtt(d), OAtt(ψ), and EAtt(Δϕ) as functions of the distance between fish d, the viewing angle ψ, and the relative heading Δϕ, for different light intensities: a-c 0.5 lx, d-f 1 lx, g-i 1.5 lx, j-l 5 lx, and m-o 50 lx (from dark to light blue). Color dots correspond to the discrete values resulting from the reconstruction procedure, extracted from the experimental data of the corresponding intensity of light. Solid lines correspond to the analytical approximation of the discrete function. Orange lines correspond to the analytical approximation of a single discrete function combining all light conditions. (PDF) [file pcbi.1011636.s027.pdf]

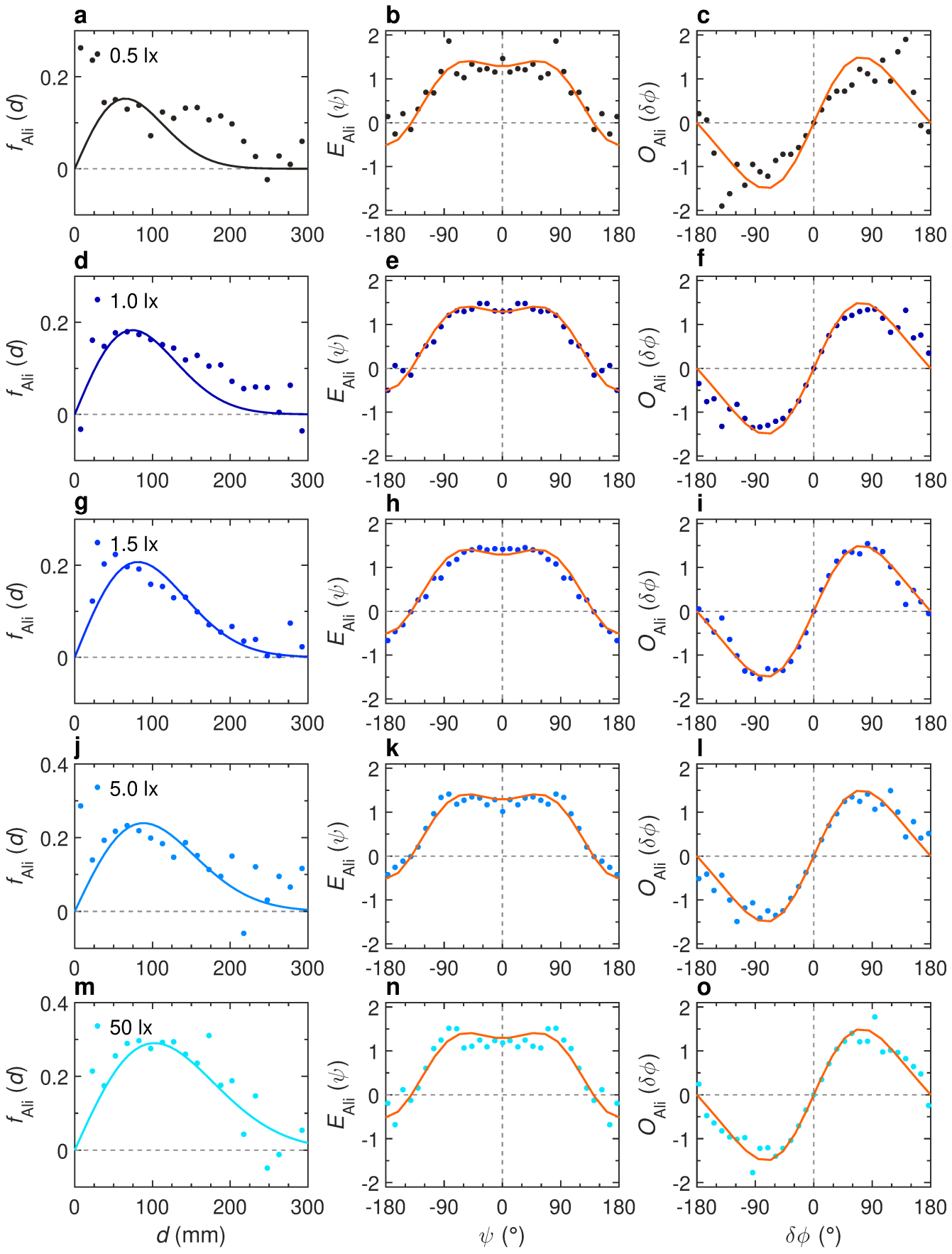

Supplement: S8 Fig — Components of the attraction interaction function fAli(d), EAli(ψ, and OAli(Δϕ) as functions of the distance between fish d, the viewing angle ψ, and the relative heading Δϕ, for different light intensities: a-c 0.5 lx, d-f 1 lx, g-i 1.5 lx, j-l 5 lx, and m-o 50 lx (from dark to light blue). Color dots correspond to the discrete values resulting from the reconstruction procedure, extracted from the experimental data of the corresponding intensity of light. Solid lines correspond to the analytical approximation of the discrete function. Orange lines correspond to the analytical approximation of a single discrete function combining all light conditions. (PDF) [file pcbi.1011636.s028.pdf]

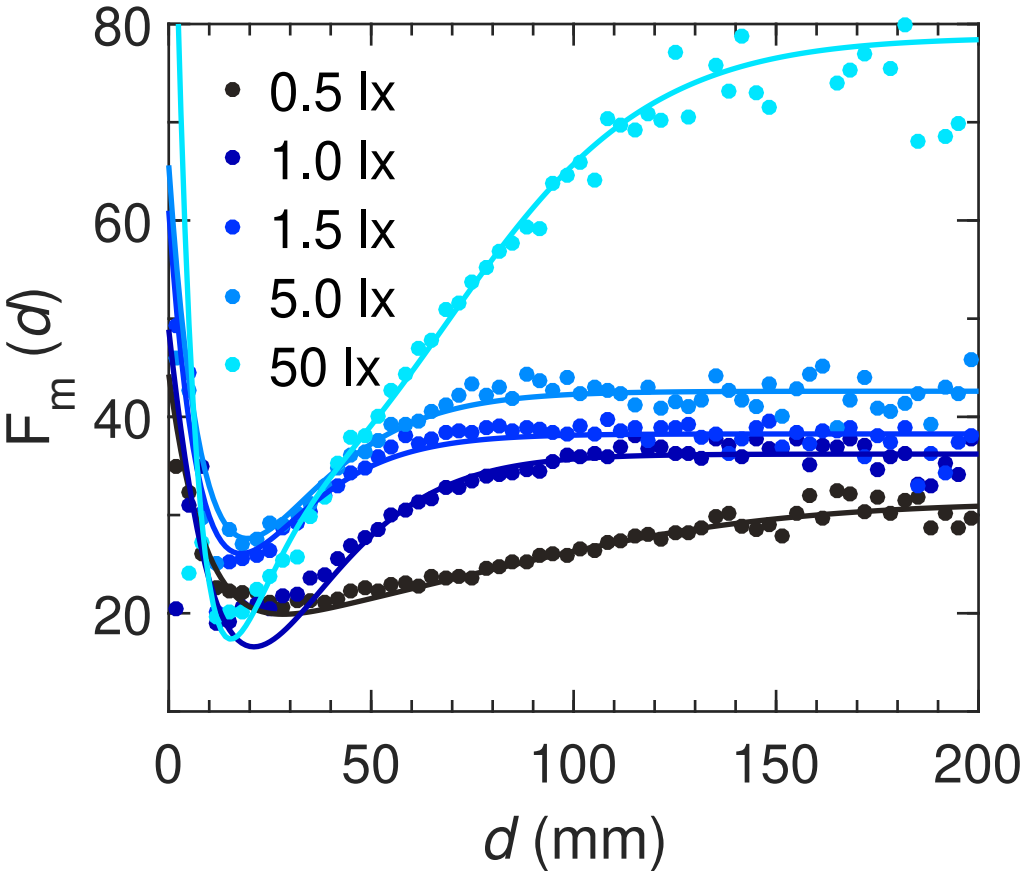

Supplement: S9 Fig — Modulation function (Fm(d)) of the mean value used in the distribution from which kick lengths are sampled, as a function of the distance between fish d, and for different light intensities: 0.5, 1, 1.5, 5, and 50 lx (from dark to light blue). Dots correspond to the discrete functions resulting from the reconstruction procedure and extracted from the experimental data. Solid lines correspond to the smooth analytical approximations of these discrete functions. (PDF) [file pcbi.1011636.s029.pdf]

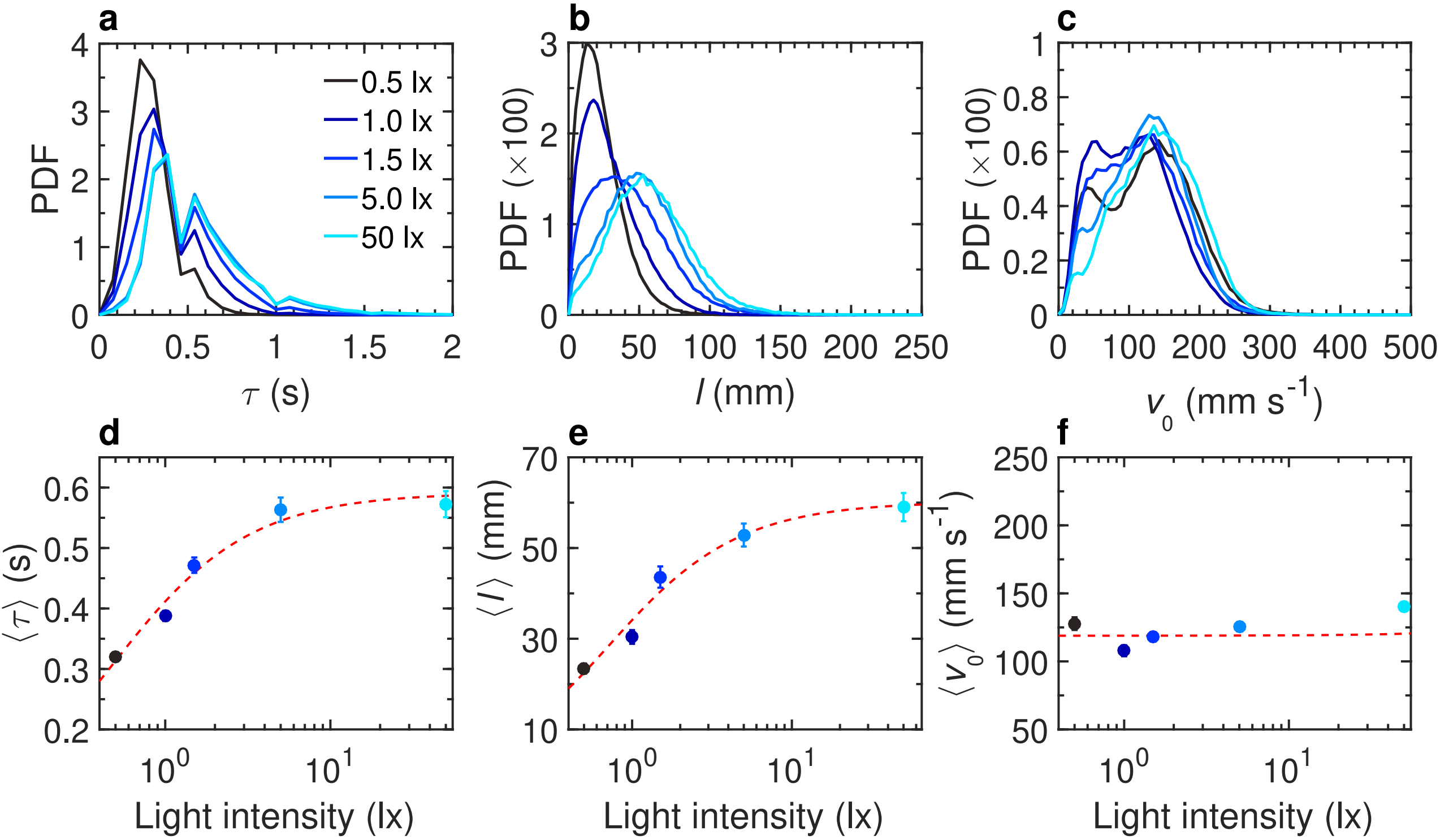

Supplement: S10 Fig — a-c Probability density function (PDF) of kick duration τ, kick length l, and peak speed v0 respectively, at different light intensities: 0.5, 1, 1.5, 5 and 50 lx (from dark to light blue). d-f Average value of kick duration 〈τ〉, kick length 〈l〉, and peak speed 〈v0〉 respectively, at different light intensities. Solid circles are the average values on all experiments; error bars represent the standard error. Red dashed lines show the trend of the average value with the light intensity. (PDF) [file pcbi.1011636.s030.pdf]

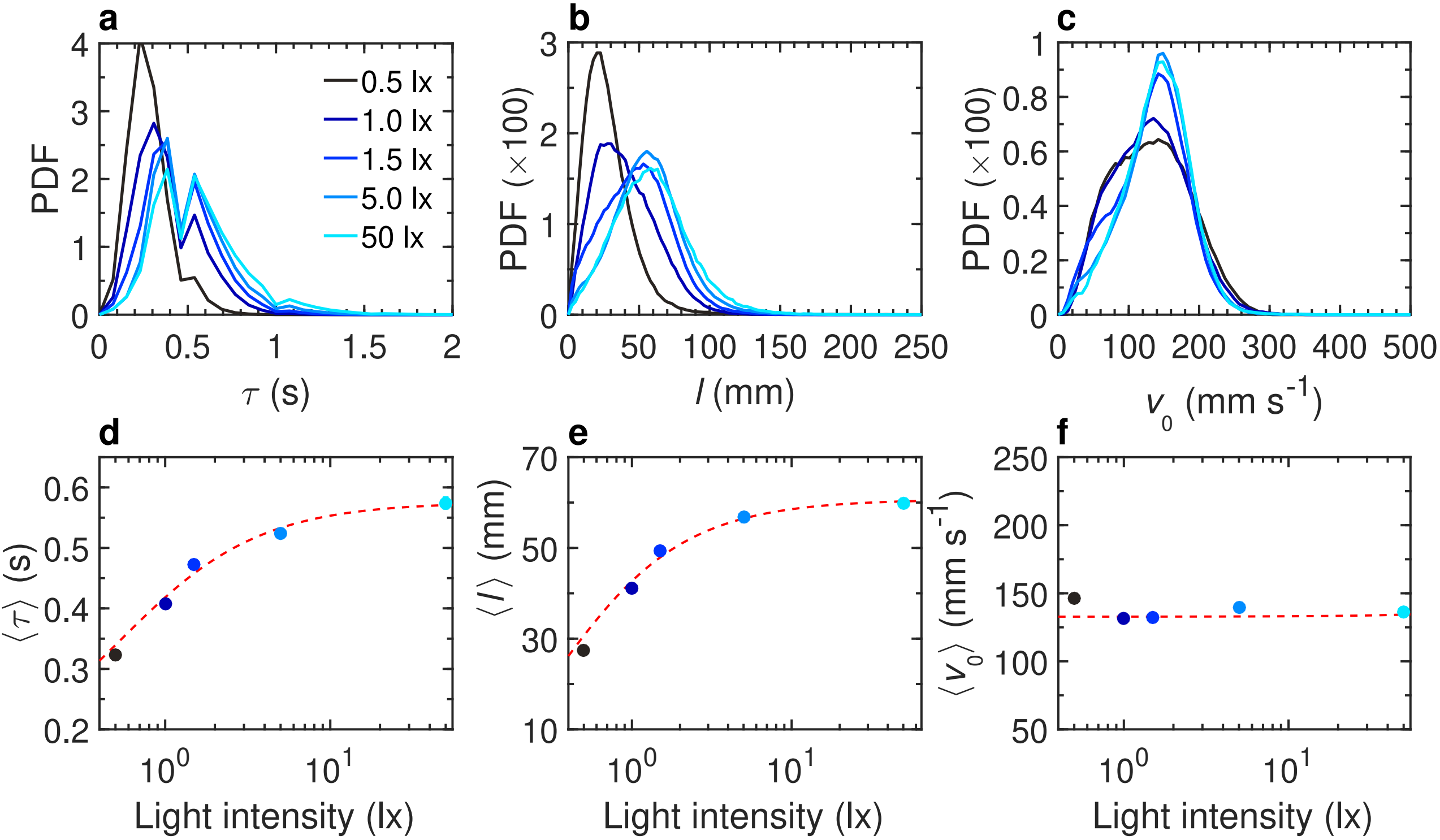

Supplement: S11 Fig — a-c Probability density function (PDF) of kick duration τ, kick length l, and peak speed v0 respectively, at different light intensities: 0.5, 1, 1.5, 5 and 50 lx (from dark to light blue). d-f Average value of kick duration 〈τ〉, kick length 〈l〉, and peak speed 〈v0〉 respectively, at different light intensities. Solid circles are the average values on all experiments; error bars represent the standard error. Red dashed lines show the trend of the average value with the light intensity. (PDF) [file pcbi.1011636.s031.pdf]

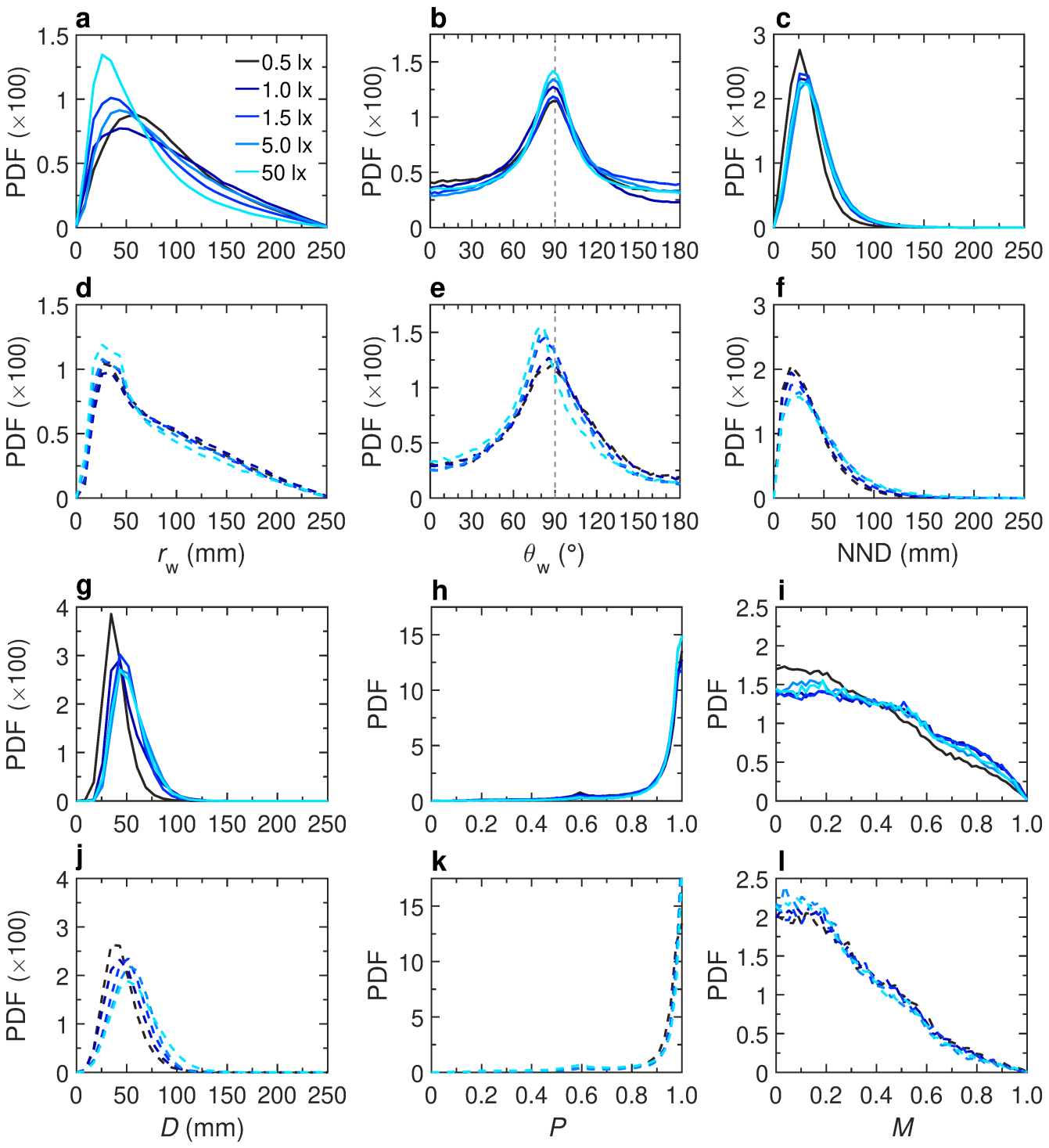

Supplement: S12 Fig — Probability density functions (PDF) of a,d the distance to the wall rw, b,e the relative angle to the wall θw, c,f the distance to the nearest neighbor NND, g,j dispersion D, h,k polarization P, and i,l milling M, for five different light intensities 0.5, 1, 1.5, 5, and 50 lx (from dark to light blue). Solid lines (a-c, g-i) correspond to experimental measures, dashed lines (d-f, j-l) to numerical simulations of the model. (PDF) [file pcbi.1011636.s032.pdf]

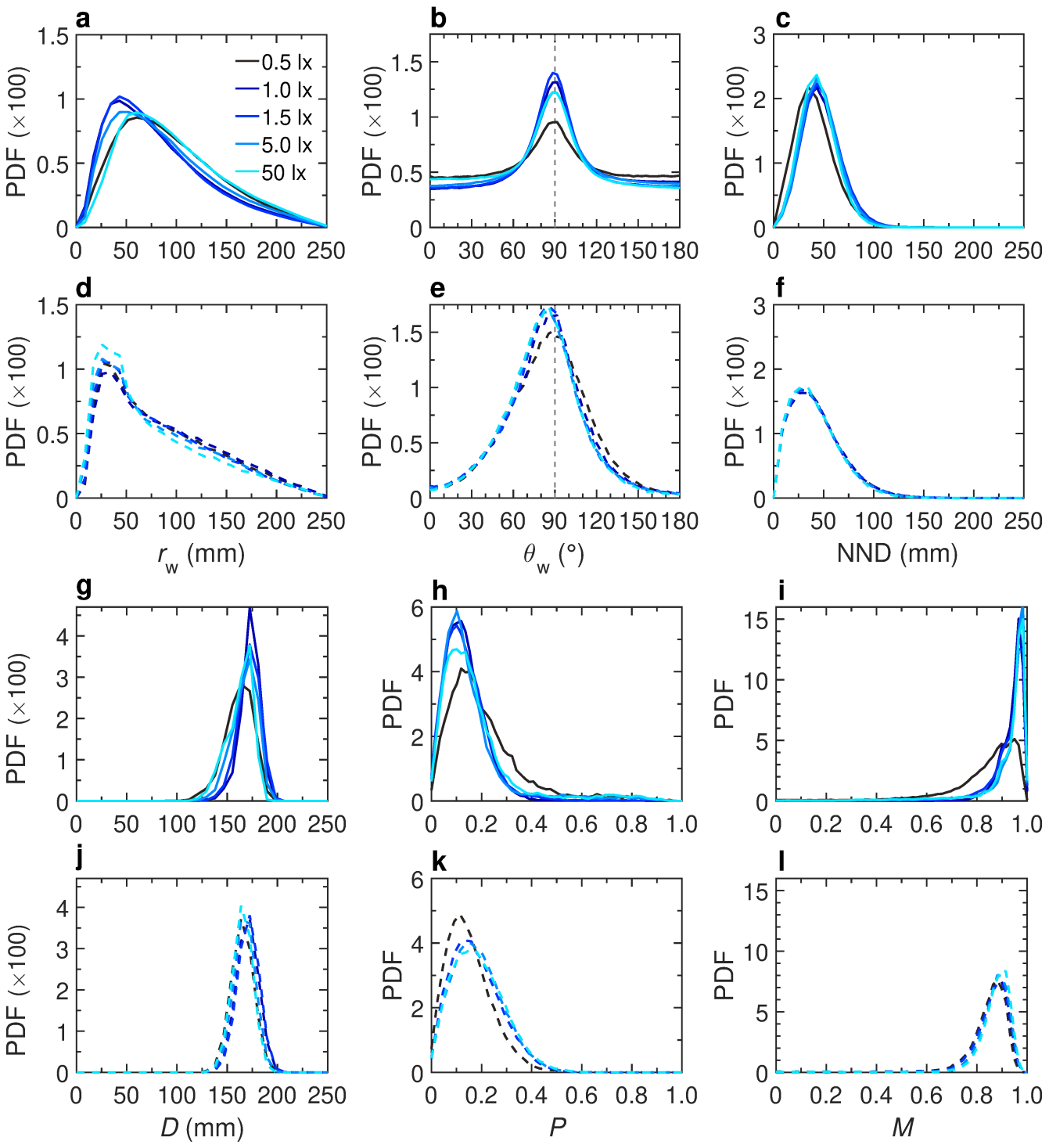

Supplement: S13 Fig — Probability density functions (PDF) of a,d the distance to the wall rw, b,e the relative angle to the wall θw, c,f the distance to the nearest neighbor NND, g,j dispersion D, h,k polarization P, and i,l milling M, for five different light intensities 0.5, 1, 1.5, 5, and 50 lx (from dark to light blue). Solid lines (a-c, g-i) correspond to experimental measures, dashed lines (d-f, j-l) to numerical simulations of the model. (PDF) [file pcbi.1011636.s033.pdf]
