# Supplementary material for: Tuning social interactions’ strength drives collective response to light intensity in schooling fish
Source: PLoS Comput Biol. 2023 Nov 17;19(11):e1011636. doi: 10.1371/journal.pcbi.1011636 (PMC10691717; doi:10.1371/journal.pcbi.1011636)
Supplement: Attachment — Submitted filename: Reply to Reviewers.pdf [file pcbi.1011636.s034.pdf]

Dear editor,

Please find attached the revised manuscript *"Tuning social interactions' strength drives collective response to light intensity in schooling fish"* by T. Xue, X. Li, G. Lin, R. Escobedo, Z. Han, X. Chen, C. Sire, and G. Theraulaz.

We would like to thank you for the opportunity to revise our manuscript and the two reviewers for their constructive reviews and for their suggestions resulting in the improvement of our manuscript.

Please find below the detailed replies to both referees and the list of changes we have made to our manuscript. We also provide a PDF file highlighting our changes (including a few minor corrections not mentioned hereafter).

Sincerely,

The authors

Reviewer's Responses to Questions

The reviewers have raised a total of 14 points that we reproduce here in black italicized text. Our answers are given in blue.

Response to Referee 1:

Here the authors have performed a valuable study that helps reveal the influence of light illumination on schooling fish. By disabling the visual system in individual fish and as a group, the authors have found that light intensity impacts fish at both the individual scale and collective scale. Interestingly, fish exhibit changes in swimming modes with changes to light intensity, which was further modeled to show predictive patterns with group sizes. The authors show that light intensity plays a large role in collective swimming and that behaviors on the individual and group scale deteriorates as the visual information with their environment decreases. Overall, the manuscript is well-written, the figures are exceptional, and the modeling work in the study are novel and improve our understanding of was how fish school, which has been a tricky behavior to study. I only suggest that the authors better introduce the study and add additional discussion of a few results.

My specific minor concerns are itemized below. The greatest of these lies in the lack of a presented motivation for this work. In the abstract and introduction, the authors should articulate how the present work makes a novel contribution to this area of investigation.

A: We wish to thank the first referee for her/his positive review and for her/his helpful and constructive comments. We appreciate that the referee considers that

the article is well written and appreciates the novelty of our experimental and modeling results. Below, we provide a point-by-point response to her/his comments and the major changes we have made to the manuscript.

***#1.1** L13:L25 – Perhaps emphasize how the current focus differs from previous papers; therefore, establishing that computational modeling can be used to predict sensory-based collective behaviors. What makes this study slightly novel and exciting is the modeling of visual cues to predict collective motion. As such, I suggest adding a sentence referring to how this study uses modeling as a way to test the visual system of schooling fish, which makes it different from other fish schooling papers.*

A: We thank the Reviewer for his/her suggestion. We added a sentence line 25 to highlight this point: *"Our computational model allows to test, quantify, and interpret - the impact of visual cues on individual and collective motion.".*

***#1.2** L26 – We hypothesize that the behavior might be multisensory, including the lateral line and vision. There has been minimal work in whether fish can use other sensory cues such as the vestibular system, separate from the lateral line, or olfaction, as seen in Pacific Salmon as a homing cue. Here, I would also cite: Mckee et al. (2020) and Mekdara et al. (2018) as they discuss that the behavior is most likely multisensory and can be species specific.*

A: We agree with this comment. We introduced the references suggested and modified the sentence as follows:

> *"In fish schools, social interactions rely on the integration of multiple sensory stimuli [46,47] including vision [43] and lateral line [44, 45], which are used to detect movements of neighbors and vibrations of the surrounding water. "*

***#1.3** L61 – After the previous paragraph, it is not clear exactly how the present study builds upon previous work. Though it's been clearly written in the discussion, I believe a few sentences justifying the importance of the present study, and explicitly stating how the study differs from others, would be useful here.*

A: We thank the Reviewer for pointing out this issue. We have modified the last paragraph of the introduction as follows:

> *"Previous studies have already investigated the role of lighting on the dynamics of collective swimming in rummy-nose tetra [McKee et al., 2020; Lafoux et al., 2023]. However, they did not analyze in this context the behavioral mechanisms and social interactions at play at the individual scale in small and large groups. Our experimental and simulation results indicate that the level of illumination does not modify the general form of social interactions between fish and their interaction with the tank wall, but only modulates the intensity and range of these interactions. Ultimately, our computational approach makes it possible to establish a direct causal link*

between (1) the modulation of these interactions by light intensity at the individual scale and (2) the specific collective motion patterns that emerge at the collective level in groups of different sizes."

#1.4 *L70:L81 & Fig. 2, 6, etc. – Were basic statistical tests for averages not done for different light conditions? Although probability density function plots and fitted curves are sufficient to show changes in data trends, effect size or how large the changes compare between groups has some value for biological significance. This is a suggestion as it doesn't change the scope/results of the paper.*

A: In lines 72-76, 92-98, and 129-148, we have explicitly added the standard errors for the different quantities mentioned (they were already reported graphically on Figs. 2, 6, S9, S11). In addition, we have performed a Wilcoxon rank-sum test for tau, l, v_0, and for N = 1, 2, 5, 25 which provides a statistical justification of our claim that tau and l are consistently increasing with the light intensity for all group sizes, whereas v_0 does not exhibit any systematic trend. These tables are reported in the new Supplementary Tables S9-S12 now also mentioned in the text (in particular on line 78).

#1.5 *L184:L187 – Do you mean that there is a tank size effect for large groups?*

A: Yes, the interactions of fish with the circular wall have a clear impact of the milling behavior observed in groups of 25 fish in comparison with the high polarization (and low milling) observed in groups of 5 fish while the distance to the nearest neighbor is similar in both group sizes. See also our answer to the comment #1.7 below.

#1.6 *Fig. 10 – I think the model being closely aligned to the experimental data is quite interesting here, especially with the polarization and milling parameters. How does the results of the model reflect the natural species-specific schooling traits? For example, what are the natural group sizes of adult H. rhodostomus and does the model reflect that?*

A: It has been experimentally shown that polarization dominates in small groups while milling dominates in large groups (McKee et al., 2020); Lafoux et al., 2023). Despite its success for aquarists, little is known about *Hemigrammus rhodostomus* since its discovery in 1924 especially regarding its ecology (Ahl, 1924). It has been reported that Rummy-nose tetras travel in groups that vary about 6 to sometimes more than 30 fish. The group sizes that we used in our experiments are of the same order of magnitude.

#1.7 *L248 – As mentioned in the above comment, is the hypothesis here that large groups no longer care about directional swimming, either with wall attraction/edge-fixation, because of the lack of motivation or is the dynamic different in larger groups? Or was it simply that the tank was too small, which reduces the*

polarization? Were there any flow cues added to the tank? The assumption here is that H. rhodostomus is a highly visual schooling-based species; and that once in larger groups, the animals no longer want to swim unidirectionally following an edge cue (the wall), especially if the visual information becomes noisier. How does the visual information change in larger groups or does it change at all since it seems that a focal fish only tracks one or two fish at a time?

A: The fact that in large groups the swimming patterns are no longer polarized, and the fish rotate around the center of the arena whatever the level of illumination is mainly a consequence of the limited size of the tank. We have modified the sentence as follows to make it clearer:

> *"In larger groups of 25 fish, the swimming patterns are no longer polarized, and the fish rotate around the center of the arena whatever the level of illumination with a high orientational milling order. This is mainly a consequence of the limited size of the tank and one can expect that without confinement, large groups would be much more polarized."*

Note that visual information used by fish to coordinate their motion does not change with group size. Each individual typically only pays attention to its two most influential neighbors. We have checked the impact of taking into account a higher number of influential neighbors with our model on the level of polarization of groups of 25 fish in the tank. Figure R1 below shows that as the number of most influential neighbors (k) with which each fish interacts with increases, groups of 25 fish become more cohesive, milling gradually decreases and polarization increases. When the number of most influential neighbors increases, each focal fish get more information about the direction of the neighboring fish, inducing a greater alignment in the moving direction of the group. The fact that we observed a low level of polarization in the experiments suggests that in large groups, fish do not change the way they pay attention to their neighbors and only interact with their two most influential neighbors.

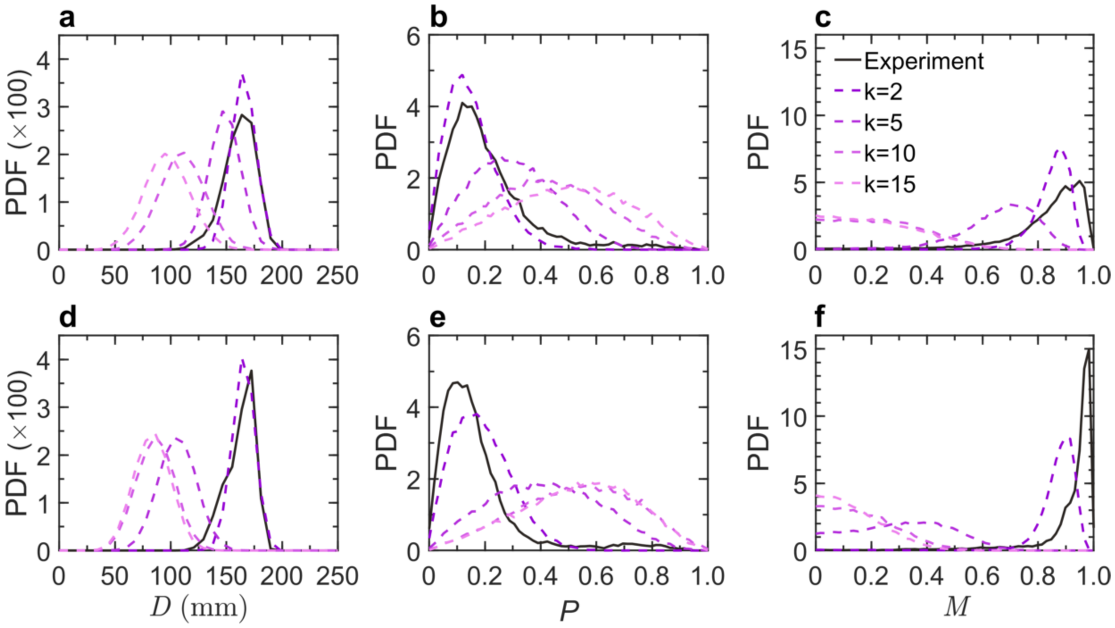

Figure R1. Effect of the number of most influential neighbors (k) on collective behavior for N=25 at 0.5 lx (a-c) and 50 lx (d-f). Probability density functions (PDF) of dispersion D (a,d), polarization P (b,e) and milling M (c,f) for different values of the number of most influential neighbors k=2,5,10,15 in the numerical simulations of the model (from deep purple to light purple) and experiments (black line).

***#1.8** L268 – There isn't a large explanation as to why the group "dispersion" parameter could be so different between n = 5 fish versus n = 25 fish. Is n = 5 fish the optimal group size under experimental conditions? What would be the optimal group size as a function of polarization and/or milling before a dramatic decrease in these parameters?*

A: Since the average distance of fish to its nearest neighbor doesn't change with group size both in experiments and simulation, the level of dispersion increases with group size simply because the total space occupied by the group is higher. We have modified the sentence as follows to make this result clearer:

> *"The model also correctly recovers the transition between a highly polarized group with a low milling rotational order for N = 5 fish, to a weak polarized group for N = 25 fish presenting a strong milling rotational order. The model also reproduces a similar average distance of fish to its nearest neighbor in both group sizes. As a consequence the total space occupied by the group increases with group size leading to a higher dispersion value."*

The group size that would maximize the polarization or milling values at least in a circular tank is an interesting question, however it is beyond the scope of this article that mainly focuses on the modulation of social interaction by light intensity.

#1.9 *L273:276 – The conclusion of the paper falls a little short as the authors make very little inferences to explain how the computation models can help predict and test the biological mechanism or the functional aspects of the system. The scope of the paper is mainly data-driven with small inferences to biology. I would like to encourage the authors to add a bit of biological context as to how the computational model can be used to examine other modes of collective behaviors seen in other species.*

A: We thank the Reviewer for his/her advice. In connection with the similar point #2.5 addressed by Referee 2 we have extended the last paragraph of the Discussion to emphasize our main findings and the potential application of our computational approach to future research on collective behavior as follows:

> *"Overall, our approach, which combines experiments with data-driven computational modeling, has allowed us to decipher how the level of illumination affects the behavior and interactions among fish, and how the modulation of these interactions at the individual level leads to changes in collective movements observed at the group level. Our results suggest that there exist robust effective interactions between fish, since only the intensity and range of these interactions, but not their functional forms, change with light intensity. This provides a general explanation for the way fish adapt their behavior and the way they interact with each other to environmental changes. Our approach that leads to an explicit and predictive model can be extended to understand and explain how the modulation of social interactions and behavior by environmental parameters (e.g., light, temperature, flow speed, etc.) or physiological parameters (e.g., stress, hunger, etc.) affects collective behaviors in animal groups."*

A: We thank the reviewer for his/her support and again for his/her comments and suggestions that helped us to improve the manuscript.

Response to Referee 2:

The authors present a systematic investigation of the impact of light intensity on the social interaction of fish by explicitly also taking into account the change in the swimming behavior of individual fish. Light intensity is the most obvious factor modulating visual perception in fish, thus it is natural to assume that it has an important impact on social interactions. Thus many studies investigated the role of light intensity, they typically were restricted to quantification of fish behavior and structure of the school without directly aiming at quantifying social interactions. In general, it is intuitive to assume that decreased light intensity, leads to weaker social interactions mediated by vision, and the previous observations seem to point into this direction. Here, the main result of the present paper, which explicitly confirms this is not really new. What is novel are the computational methodological aspects, of explicitly mapping out and fitting the interaction functions for the various contributions governing fish swimming behavior (interaction with the wall, and social

interactions attraction+alignment) at different light intensities, using a general methodology established previously by the same group. What the results show that the functional shape of the interactions does not change with the light intensity, which in principle could also be the case. To my knowledge this is the most in-depth and systematic investigation of these aspects up to date, and thus of potential interest to a broader audience interested in collective behavior, fish behavior and visual ecology more generally. Importantly, the authors combine their experimental work with simulations of an individual-based model to test how far the observed changes in social interactions, can explain also the emergent patterns of collective swimming.

The paper is well written, the figures are of good quality thus, I think the work if potentially suitable for publication with PLoS Comp Biol. However, there is a number of aspects, I would expect the authors to consider / revise.

A: Before we address the reviewer's comments point-by-point, we would like to thank his/her positive evaluation of the manuscript.

#2.1 *Individual swimming: The authors state that the spontaneous heading change away from walls is Gaussian distributed. While this seems to be (at least approx.) the case for the larger light intensities. The results in Supp. Fig 3, clearly show deviations from a Gaussian for smaller intensities (<=1.5lx). Here it seems to be more exponentially distributed. I expect this to be correctly reported and maybe also discussed in the context of other experimental findings.*

A: We thank the referee for his (her) comment. Indeed this point requires some clarification. First, let us mention that the main point of Fig. S3 was to quantify the increase and ultimate saturation of the standard deviation of the spontaneous heading fluctuations of the fish by using the *same* model (Gaussian) PDF for all lights, for consistency. As pointed out by the referee, the Gaussian fits are better in high light condition than in low light conditions. There are reasons for this result: to compute individual random headings, we selected the data where fish were far away from the wall. As can be seen in Fig. 3a, the distance between the fish and the wall is basically around 20mm, and there are very few available data when fish are away from the wall (i.e. when r_w > 60mm). In addition, from the distribution shown on Supplementary Fig. 3, one can observed that the random headings are relatively small, basically within the range of [-20°, 20°], and the fitting effect of Gaussian distribution in this interval is quite good.

Certainly, following your suggestion, we also try to fit the data with an exponential distribution, as shown in the Figure R2 below. The dashed lines represent the Gaussian distribution fit, while the solid lines represent the exponential distribution fit. We believe that the overall fitting results are indeed quite good, especially under low light intensity conditions. However, under high light intensity, in the region of

interest (-20°, 20°), especially close to 0°, the Gaussian distribution fits better. For PDF> 5*10^-3, we can see that the Gaussian distribution almost perfectly captures the pattern.

Ultimately, we consider that the Gaussian distribution provides a satisfactory fit of the distributions in the region of high probability. Moreover, the fit to the *same* distribution for all lights allows us to determine a consistent intensity of the spontaneous heading fluctuations with a reasonable accuracy.

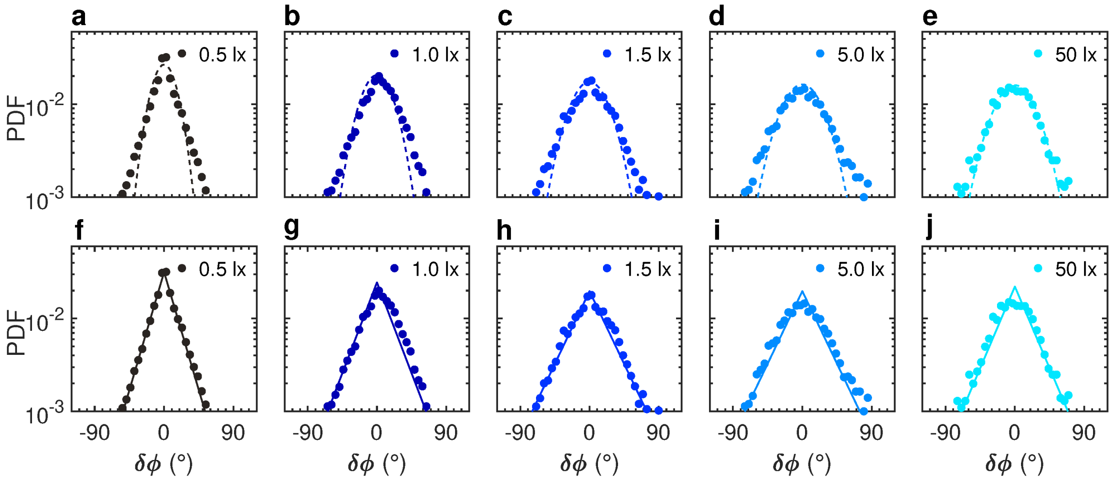

Figure R2. Effects of light intensity on the spontaneous heading change of a fish swimming alone (N = 1). The dashed lines represent the Gaussian distribution fit, while the solid lines represent the exponential distribution fit.

#2.2 *The caption of Supp Fig 3 it states that solid lines are the approximation with a Gaussian distribution. However, the lines are dashed.*

A: Thank you for pointing out this error. We have corrected it.

#2.3 *Interactions with the wall: For the largest light intensities 5.0 and 50lx there appears to be clearly an attractive force to the wall above 70mm. Which the authors seem not to comment on / discuss.*

A: As shown on Figure 3, the PDF of the distance r_w to the wall has very little weight for r_w > 60 mm. For the 5 light intensities from 0.5 to 50 lx, we find that the residual percentage of data for r_w > 60 mm is respectively 12.7%, 8.4%, 7.0%, 4.2%, 3.1%, while for r_w > 80 mm, we find respectively 8.1%, 4.2%, 3.6%, 1.8%, % 1.3%. Hence, the interaction with the wall (Fig. 4) for, say, r_w > 80 mm is ill-determined as illustrated by the large amplitude of the fluctuations observed in Fig. 3. Although one cannot exclude a small attractive component of the interaction with the wall at large distance for light intensity 5 and 50 lx, its relative magnitude would be anyway much smaller than for r_w < 20 mm where the weight of the PDF of r_w is concentrated for all light intensities. We have added a short remark in the legend of Fig. 4, clarifying the fact that the interaction with the wall is ill-determined for distances to the wall typically above 80 mm, due to the lack of data there.

We have also added a few sentences in the paragraph ‘Modeling and measurement of fish interaction with the wall in different light conditions’ to highlight this point and also the next point #2.4:

> *“For high light intensity, there are significant deviations between the fits of $f_{\rm w}(r_{\rm w})$ and $O_{\rm w}(\theta_{\rm w})$ and the actual data points, which indirectly confirms that individual movement patterns are indeed different under varying light intensities. Fish prefer to stay closer to the walls and to move in directions parallel to the walls under high light intensity (see Fig~\ref{fig3}). This leads to a concentration of the data distribution, with relatively few data points available when fish are far from the wall and non-parallel to the wall. Consequently, the reconstruction of the interaction with the wall is reasonably precise in the regions of $r_{\rm w}$ and $\theta_{\rm w}$ associated to a high probability, but exhibits large fluctuations for $r_{\rm w}>80$\,mm or $\theta_{\rm w}$ far enough from~$\pm 90^\circ$.”*

***#2.4** Furthermore, what is interesting is that also the fit of the angular dependence seems to become worst at maximal light intensity of 50lx. I think this is at least worth to mention as well as well as potentially discuss.*

A: Similarly, we face the same issue with the fitting of O_w, because as the light intensity increases, fish tend to move more in directions away from the walls (θ_w is close to 90°; see our reply to point #2.3). This results in a concentration of data in some areas, leading to larger fluctuations in the fitting in other regions. Moreover, as light intensity increases, the fitting errors become more significant.

***#2.5** Discussion general findings: I think the result that the functional form of the interactions does not change with the light intensity, should be highlighted more. As this is not trivial, as in principle the functional form could also change due to constraints in vision. The results suggest that there is somehow a robust effective interaction between the fish, where only the strength is modulated.*

A: We thank the Reviewer for his (her) advice. You are right this result must be emphasized in the discussion. We added the two following sentences in the Discussion to emphasize our main findings:

> *“Our results suggest that there exist robust effective interactions between fish since only the strength and range of these interactions but not their functional forms change with light intensity. This provides a general explanation for the way fish adapt their behavior and the way they interact with each other to environmental changes.”*

We thank Referee 2 for his (her) comments that help convey our findings more clearly. We hope to have clarified his (her) concerns in the revised version of our manuscript.
